# Regulatory Mechanisms of Silver Nanoparticles on Seed Germination: A Multilevel Integrative Perspective

**DOI:** 10.3390/ijms27041692

**Published:** 2026-02-10

**Authors:** Yawen Zheng, Chongyuan Qin, Peilin Han, Yinuo Pan, Yingxin Han, Hengjin Chen, Xiumei Wang, Juanxia Li, Jixiang Lin, Jinghong Wang, Lirong Zhang

**Affiliations:** 1 College of Landscape Architecture, Northeast Forestry University, Harbin 150040, China; 2024120574@nefu.edu.cn (Y.Z.); han-peilin@nefu.edu.cn (P.H.); 2024120588@nefu.edu.cn (Y.P.); hanyx@nefu.edu.cn (Y.H.); chenhengjin@nefu.edu.cn (H.C.); wxiumei@nefu.edu.cn (X.W.); ljxyuanlin@nefu.edu.cn (J.L.); linjixiang@nefu.edu.cn (J.L.); 2 Guangxi Key Laboratory of Plant Conservation and Restoration Ecology in Karst Terrain, Guangxi Institute of Botany, Guangxi Zhuang Autonomous Region and Chinese Academy of Sciences, Guilin 541006, China; chongyuanqin@gxib.cn; 3 Chinese Academy of Environmental Planning, Beijing 100012, China

**Keywords:** silver nanoparticles (AgNPs), seed germination, molecular mechanisms, nanofertilizers, plant physiology

## Abstract

With the growing global population and the challenges posed by climate change on agriculture, improving seed germination quality has become an urgent task. Nanotechnology, particularly silver nanoparticles (AgNPs), offers a promising approach to this issue. However, their long-term environmental impact and health risks require further evaluation.This review first explores the physicochemical properties of AgNPs and their effects on plant growth and seed development. Next, the review discusses the mechanisms by which AgNPs enhance seed resistance to pathogens, regulate reactive oxygen species (ROS) balance, activate key metabolic enzymes, induce metabolite accumulation, and modulate plant hormone levels. Additionally, the review explores how AgNPs influence seed gene expression, proteomic networks, and the germination microenvironment. Given the lack of field data on long-term low-dose exposure and challenges in monitoring morphological transformation, the review also evaluates the potential risks of AgNPs in agriculture. These risks include their accumulation in the food chain, environmental transformation, and long-term effects.The review aims to summarize the mechanisms by which AgNPs impact seed germination and plant growth, providing a theoretical basis for their cautious use in agricultural and horticultural practices, while considering their environmental fate and health risks.

## 1. Introduction

Under the dual pressures of a continuously growing global population and intensifying climate change, agricultural production urgently requires innovative technologies to enhance crop stress resistance and yield. Silver nanoparticles (AgNPs), with their unique physical and chemical properties—such as their strong adsorption capacity due to their high specific surface area [1], the transmembrane penetration ability conferred by their nanoscale size, and their exceptional antimicrobial properties [2]—are gradually emerging as a novel tool in the agricultural sector. Currently, studies have found that AgNPs can influence the genes of aquaporins in seeds, thereby increasing water uptake and promoting earlier germination [3]. By enhancing the activity of antioxidant enzymes in seeds, AgNPs can stimulate seeds to improve stress resistance and bolster their tolerance to adverse conditions [4]. Meanwhile, AgNPs also have an inhibitory effect on bacterial growth, protecting seeds from pathogen damage, thus preventing seed rot and safeguarding seed integrity [5].

However, despite the considerable attention on the potential of AgNPs in agricultural applications, the mechanisms underlying their effects on seed germination remain controversial and poorly understood. Existing research indicates that the impact of AgNPs on seed germination exhibits a dose-dependent effect: at low concentrations, AgNPs may promote germination by regulating internal redox balance and activating germination-related enzyme activities, whereas high concentrations of Ag^+^ and AgNPs can induce oxidative stress, inhibit seed respiratory metabolism, and lead to a decline in germination rate [3]. Furthermore, significant differences exist in the sensitivity of different plant species to AgNPs, and distinct variations in responses are also observed among different ecotypes or cultivars of the same species. These factors pose substantial challenges to the standardized application of AgNPs in agricultural production.

Current studies primarily focus on single indicators, such as germination rate and radicle length, under laboratory conditions. They lack a systematic exploration of AgNPs’ effects in real agricultural environments. Furthermore, research on environmental interaction effects, such as how soil pH and microbial communities influence AgNP activity, is limited. There is also insufficient investigation into long-term ecological risks, including the impact of AgNP accumulation in soil on subsequent crops. Furthermore, several studies have used citric acid or chitosan-modified nanoparticles in seed treatment. For instance, chitosan nanoparticles significantly enhanced germination rate, seedling vigor, and resistance to fungal diseases in rice, tomato, pearl millet, and maize seeds [6]. Additionally, citric acid-coated nanoparticles significantly enhanced the germination rate and promoted seedling growth in radish seeds [7]. However, the molecular mechanisms by which the particle size, surface modification materials, and silver ion release of AgNPs synergistically regulate seed germination remain to be elucidated.

The existence of these scientific issues severely restricts the transformation process of AgNPs from laboratory research to practical agricultural applications. Although AgNPs exhibit potential in promoting germination and enhancing stress tolerance in agriculture, experimental studies have shown that when ingested orally by mice, silver can accumulate in the liver, kidneys, and brain, suggesting potential neurotoxic and reproductive risks. Moreover, once AgNPs enter the environment, they can biomagnify in the food chain, inhibiting the survival, reproduction, and microbial balance of soil and aquatic organisms. The difficulty in monitoring their morphological transformation poses long-term, uncertain risks. Currently, there is a lack of data on low-dose, long-term field exposure and insufficient research on the morphological forms of AgNPs in humans and animals [8]. Therefore, before integrating AgNPs into agricultural practices, a systematic assessment of their environmental fate and health risks is required. This is to prevent the “technological solution” from turning into a new pollution issue.

**Figure 1:**
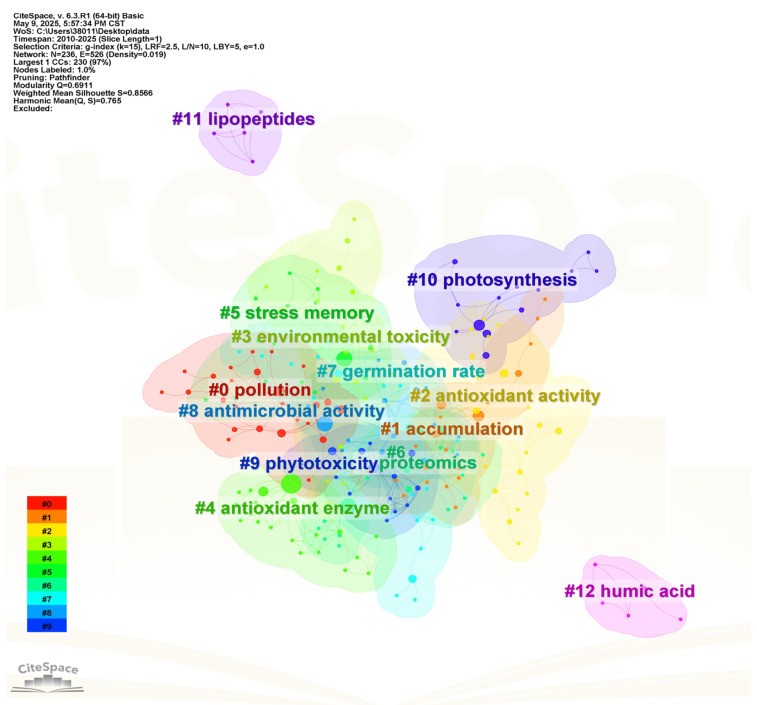
Cluster diagram of keywords in the research field of the interaction of AgNPs with seed germination.

Therefore, the core focus of this review is on the unique mechanisms of AgNPs, their dose-dependent thresholds, and molecular signaling networks. Specifically, we systematically analyze how the physicochemical properties of AgNPs, such as concentration and particle size, regulate seed germination dynamics and physiological responses. We also explore the molecular and biochemical pathways affected by AgNPs, including the regulation of reactive oxygen species (ROS) homeostasis, activation of metabolic enzymes, and reprogramming of plant hormone balance. Additionally, this review evaluates the ecological risks and long-term effects of AgNPs in agricultural ecosystems, highlighting the urgent need for further research to clarify their environmental persistence and potential toxicity. Over the past 15 years, there has been a growing number of studies on the Interaction of AgNPs with seed germination. Literature retrieval was conducted using the Web of Science (WoS) Core Collection database, and CiteSpace (Version 6.3) was employed for visual analysis of the literature. Keyword analysis (Figure 1) yielded 12 clusters, with a Q-value of 0.6911 (greater than 0.3, indicating a significant clustering effect) and an S-value of 0.8433 (greater than 0.7, demonstrating a distinct clustering structure) [9]. As observed from the keyword co-occurrence clustering map, the WoS keyword clusters include the following: “pollution”, “accumulation”, “antioxidant activity”, “environmental toxicity”, “antioxidant enzyme”, “stress memory”, “proteomics”, “germination rate”, “antimicrobial activity”, “allium cepa”, “photosynthesis”, “lipopeptides”, and “humic acid” (Figure 1).

**Figure 2:**
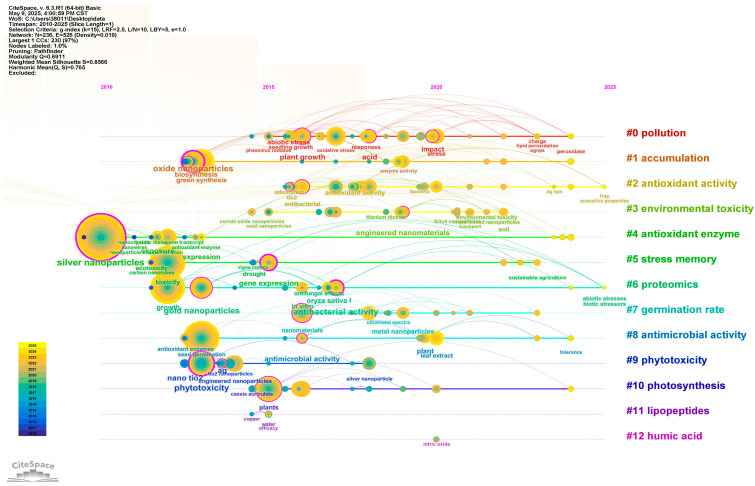
Timeline Chart of Keywords in AgNP Research on Seed Germination.

By aligning the keywords of the same cluster on a single axis, a timeline visualization of the changes in these cluster keywords can be generated (Figure 2), where a larger node indicates a higher frequency of the corresponding keyword. During the 10-year period from 2010 to 2020, the “antibacterial activity” of AgNPs (either alone or in combination with other nano-metals or metal oxides) on “plant growth”, along with “environmental toxicity” and “abiotic stress”, emerged as research focuses. Subsequently, topics such as “peroxidase”, “charge”, “transport”, and “sustainable agriculture” have become hot issues in recent years (2020–2025) (Figure 2).

**Figure 3:**
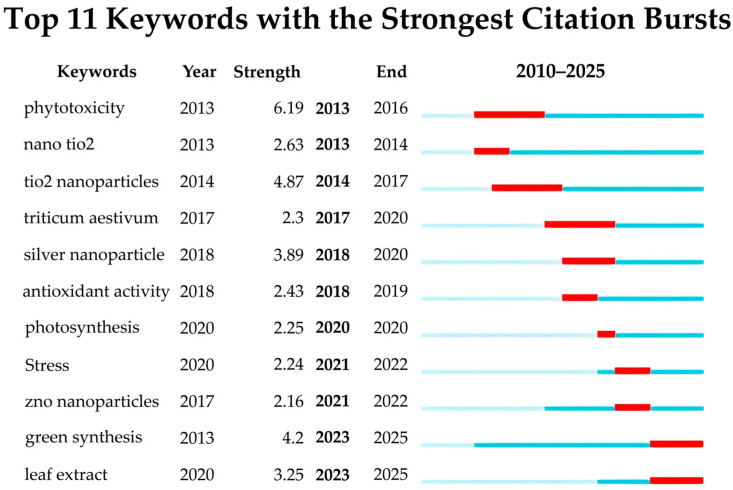
Burst Keywords in AgNP Research on Seed Germination.

The burst keywords in the field of research on the interaction of AgNPs with seed germination over the past 15 years have been organized, as shown in Figure 3. In the most recent two years, there has been a growing focus on “leaf extract” and “green synthesis”, indicating that more optimal and environmentally friendly synthesis methods of AgNPs are among the current research hotspots. Meanwhile, research has mainly concentrated on the effects of AgNPs alone, as well as their mixtures with other nanomaterials such as “nano TiO_2_”, on seed germination under stress conditions (Figure 3).

In recent years, the continuous deterioration of the ecological environment and the increasing human demand for food and ornamental plants have drawn significant attention to the efficiency and quality of seed germination. In light of this, this paper reviews and summarizes domestic and international research progress on the physiological mechanisms by which AgNPs affect seed germination. The review is conducted from two perspectives: the growth and physiological impacts of AgNPs on seed germination. It aims to clarify the mechanisms underlying the Interaction of AgNPs with seed germination, thereby providing a reference for the future application of AgNPs in agriculture and forestry to promote plant growth. Additionally, it proposes future research directions, intending to offer a theoretical basis for further exploration of the regulatory mechanisms of AgNPs on seed germination.

## 2. Physicochemical Properties of AgNPs and Seed Priming Methods

The particle size of AgNPs generally ranges from 1 to 100 nm. Compared with traditional chemical synthesis methods, biosynthesis-based preparation technologies for metal nanoparticles (NPs) stand out due to their environmentally friendly characteristics. During the biosynthesis process, biological extracts can enhance the stability of nanoparticles through diverse synthesis conditions and technical approaches [10,11]. Table 1 presents the particle size, characterization, and stability of some biosynthesized AgNPs, as well as their effects on seed germination.

Through the table, it can be observed that AgNPs synthesized by biological methods generally exhibit the best stability. This is primarily attributed to the encapsulation effect of natural organic compounds, which effectively protect the AgNPs from aggregation or degradation. Additionally, this process enhances their biocompatibility and environmental stability [12]. In contrast, AgNPs synthesized using plant extract methods also exhibit significant stability, especially under conditions of increased pH. This stability is notably improved due to the enhanced surface negative charge and the reduction in particle size [13]. This process reflects the critical impact of nanoparticle surface charge on their dispersibility and stability. Although chemical synthesis methods can produce high-purity AgNPs, their stability is relatively lower compared to biological and plant extract methods. This is especially true in the absence of appropriate surface modifications, as the particles tend to aggregate or oxidize.

Using AgNPs coated with stabilizers, such as hyaluronic acid (HA) [14], can effectively improve seed germination rate and vigor index. In this context, the amine (NH_2_) and carboxyl (–COOH) functional groups in ALA interact with metallic Ag through electrostatic interactions, acting as capping agents to enhance the stability of AgNPs. Another method for AgNP priming involves preparing a suspension of AgNPs in distilled water through ultrasonication, followed by addition to the Murashige and Skoog (MS) medium containing silver nanoparticles. After autoclaving the medium and allowing it to solidify, seeds can be sown directly [15]. Notably, uncoated AgNPs exhibit high surface energy and tend to show poor stability in certain media [16], where they are highly prone to aggregation [17], thereby reducing the efficacy of AgNP applications. Therefore, capping agents are commonly used in experiments to coat AgNPs in order to improve their stability. For example, polyvinylpyrrolidone (PVP)-coated AgNPs (AgNP-PVP) can enhance their colloidal stability in culture media [16]. In a study on AgNP priming of tobacco seeds, AgNP-PVP and cetyltrimethylammonium bromide (CTAB)-coated AgNPs (AgNP-CTAB) were separately added to sterilized MS nutrient medium. The results indicated that the surface plasmon resonance (SPR) peak position and absorbance of AgNP-PVP underwent significant changes, whereas AgNP-CTAB demonstrated better stability in the MS medium, while AgNP-PVP was relatively more susceptible to dissolution and aggregation [18].

**Table 1:** Differences in Properties of AgNPs Prepared by Different Synthesis Methods and Their Effects on Seed Germination.

Synthesis Method	Particle Size	Characterization	Stability	Reference
Under light-excluded conditions at 45 °C and pH 9, the mycelial filtrate of the medicinal plant endophytic fungus *Penicillium polonicum* PG21 reacted with 13,590.4 mg/L AgNO_3_ for 48 h.	3–25 nm	Spherical.	The proteins secreted by *Penicillium polonicum* PG21 are adsorbed onto the surface of the newly formed AgNPs, forming a coating layer. This coating enhances the stabilization of AgNPs and prevents aggregation.	[19]
Aqueous extracts of two cyanobacteria (*Westiellopsis ramosa* and *Nostoc commune*) were prepared separately and mixed with 169.9 mg/L in a 1:1 ratio. The reaction was conducted at 50 °C and pH 6–8 for 15–30 min, during which the solution changed from colorless to brown.	The average particle size of the *Westiellopsis* derived product was 57.6 nm, while the average particle size of the *Nostoc* derived product was 46.6 nm.	Spherical.	During the biosynthesis of cyanobacteria, while reducing Ag^+^ to AgNPs, the proteins, polysaccharides, and other biomolecules in the extract act as natural capping agents, coating the particle surface. This significantly inhibits aggregation and oxidation, allowing the silver nanoparticle colloid to remain stably dispersed over a long period.	[20]
AgNPs were synthesized by adding neem (*Azadirachta indica*) leaf extract to a silver nitrate solution, stirring at 80 °C for 10 min, while adjusting the pH of the extract (5.7, 7, 8.5, and 10).	pH 5.7:142.8 nm; pH 8.5:51.75 nm; pH 10:22.04 nm (all values are average particle sizes)	Spherical or ellipsoidal	AgNPs synthesized at pH 8.5 showed a zeta potential of −23.73 ± 0.54 mV, with a relatively high absolute value, indicating strong negative surface charge that provides electrostatic repulsion to prevent aggregation and maintain stable dispersion.	[21]
1.69 × 10⁴ mg/L AgNO_3_ solution was added dropwise (1 drop/s) into 10 mL of *Nelumbo nucifera Gaertn.* leaf extract, followed by magnetic stirring at room temperature for 3 h.	Average size: 12.87 nm, range: 4–24 nm	Spherical or nearly spherical	The zeta potential of AgNPs was −28.6 mV. The negative potential indicates that the negatively charged components in the lotus leaf extract act as capping agents, providing electrostatic repulsion to maintain stable dispersion of AgNPs in aqueous solution.	[22]
AgNPs were synthesized by mixing 10 mL of plant extracts (from *Azadirachta indica* or *Moringa oleifera* leaves) or fungal extract (from *Aspergillus niger*) with 50 mL of 169.9 mg/L AgNO_3_ solution, stirring at 25 °C for 20 min, and then kept in the dark until the solution color changed from red to dark brown.	25–45 nm	Spherical, showing a polydisperse distribution	TEM (transmission electron microscope) observations revealed that AgNPs were relatively small in size and uniformly distributed, with no obvious aggregation, indicating good stability.	[23]

Compared with the aforementioned methods, there are relatively fewer literature reports on AgNP-based seed coating technology. This technology involves uniformly adhering AgNP treatment solution to the seed surface by stirring with a stir bar or manual stirring, and then placing the treated seeds at room temperature to dry naturally. Silver nanoparticles–chitosan (AgNPs-CH) can be used as a seed coating agent; its chitosan layer provides Coulombic forces to offset the van der Waals attractive forces between nanoparticles [24], significantly enhancing the stability of AgNPs. Studies have shown that AgNP-CH, as a seed coating agent, exhibits no obvious toxicity to plant growth, can improve seed germination traits, and thus can serve as a safe seed coating agent for preventing early fungal infections of seeds [25]. Seed coating is a promising agricultural technology, yet data on potted and field applications of AgNP-based seed coating remain relatively scarce.

## 3. Interaction of AgNPs with Seed Germination

During seed germination, seeds are often affected by both biotic and abiotic stresses. For instance, seeds are also affected by biotic factors such as fungi and blight. When seeds resist the stress from pathogenic microorganisms, they produce more abscisic acid (ABA), which disrupts the balance between ABA and gibberellin (GA) levels and inhibits seed germination [26]. Under different types of stress, AgNPs also have a significant promotive effect on seed germination. Studies have found that soaking seeds in AgNP solution or culturing seeds in a medium containing AgNPs exerts a significant impact on seed germination: AgNPs can act as an antibacterial agent, effectively inhibiting biotic stresses caused by microorganisms on seeds and improving seed integrity [27]. Under heat stress at 32.2 °C, potato seeds soaked in 5 mg/L AgNP solution showed increased germination rates and enhanced root growth speed [28]. Under 8.78 × 10^3^ mg/L NaCl salt stress, the germination rate of hard red wheat seeds soaked in 140 mg/L AgNP solution increased from 70% to 90%, with significant improvements in root length, root fresh weight, and root number. This suggests that AgNPs can effectively alleviate the inhibitory effects of salt stress on seed germination and root growth [29]. Furthermore, under industrial wastewater stress, AgNPs significantly improved the germination rate of maize seeds, demonstrating their effectiveness in alleviating heavy metal stress [30]. In the absence of stress, AgNPs can increase the number of germinated wheat seeds and the fresh weight of the above-ground parts and roots of wheat seedlings [31]. However, the interaction of AgNPs with seed germination exhibits significant pleiotropy and complexity. For example, although radish seeds soaked in AgNP solution can still germinate normally, root elongation nearly stops, the root tips show brown necrosis, and lateral roots are reduced. This indicates that the toxic effects occur in the newly formed root tissues rather than during the germination process [32]. Therefore, a thorough analysis of the interaction of AgNPs with seed germination is a key prerequisite for revealing their mechanism of action and promoting the safe application of AgNPs in agriculture.

### 3.1. The Effects of Concentration and Particle Size on Seed Germination

The interaction of AgNPs with seed germination exhibits a concentration window effect, meaning that the same species may show different responses at different AgNP concentrations. This phenomenon reflects the existence of distinct “safety thresholds”. When wheat seeds were placed in Petri dishes with varying concentrations of AgNPs (10–40 mg/L), the germination rate remained favorable. However, at concentrations ≥ 20 mg/L, AgNPs significantly reduced the total identified polar metabolites (TIPMs) in the endosperm, with glucose, fructose, and galactose showing the greatest decline. At the same time, the total amino acid (TAA) and organic acid (TOA) contents decreased as the concentration increased, while starch and storage protein mobilization were inhibited. This led to a reduction in seed osmotic potential, a decrease in water potential gradient, and a suppressed imbibition rate, exhibiting dose-dependent phytotoxicity [33]. Another study showed that wheat seeds were soaked in 10, 25, 50, 100, 200, and 300 mg/L AgNP solutions. Only the 200 and 300 mg/L AgNP treatments significantly increased plant height, root length, and chlorophyll content in wheat seedlings. The experiment inferred that AgNP treatment formed small pores on the seed coat and generated N-amylase complexes, which promoted starch hydrolysis, resulting in high levels of sugars. The increased cellular osmotic pressure enhanced water uptake by the seeds, promoted embryonic development, and thereby increased growth and germination rates [34]. Therefore, it is evident that the promotion and inhibition of seed germination by AgNPs in the same plant species occur within a narrow concentration window.

Furthermore, the interaction of AgNPs with seed germination exhibits a typical concentration-dependent effect. During the imbibition stage of pea seeds, they were soaked in aqueous solutions of different concentrations of AgNPs. The results showed that treatment with 270 mg/L AgNPs increased the germination rate of pea seeds and enhanced leaf number, stem and root length, dry weight, and fresh weight, while causing no harm to symbiotic microorganisms in the soil. In contrast, 108 mg/L and 540 mg/L treatments inhibited pea growth [35]. Another study showed that seed treatment with AgNPs at concentrations of 20, 40, and 80 mg/L significantly enhanced the root length, fresh weight, and leaf yield of Chinese cabbage seedlings. Inductively coupled plasma-mass spectrometry (ICP-MS) analysis revealed that the Ag content in the seeds after treatment with 20, 40, and 80 mg/L was 3.3, 14.9, and 7.3 mg/kg, respectively, which was significantly higher than the control. This confirmed that AgNPs or the released Ag^+^ were absorbed into the seed embryo during the hydration phase. However, based on the germination rate data, concentrations of 20–40 mg/L significantly accelerated seedling emergence and improved seedling quality, while 80 mg/L delayed germination in the early stages. Therefore, the optimal concentration was 20–40 mg/L [36].

The particle size of AgNPs can also influence the fate of seeds. At the same concentration, different particle sizes have a significant impact on the seed metabolic profile. After treatment with 20 mg/L citrate-coated AgNPs (except for the 60 nm particle size), there was a slight decrease in the TIPMs in 3-day-old wheat coleoptiles. At the same concentration, as the particle size increased from 10 nm to 100 nm, the total amino acids (TAAs) and total organic acids (TOAs) in the coleoptiles showed a slight decreasing trend. When the particle size ranged from 20 to 60 nm, sucrose concentration in both the root and coleoptile increased significantly, while the contents of monosaccharides such as glucose and fructose decreased accordingly [37].

### 3.2. AgNPs, Seed Germination Kinetics, and Associated Physiological Changes

During the seed germination process, porosity, water potential, and the driving force for water uptake are all important influencing factors, and they play a key role in the water uptake efficiency of seeds. Porosity often determines the water penetration capacity of seeds; higher porosity accelerates the entry of water into the interior of seeds [38], promotes internal metabolic activities, and thereby indirectly affects water potential and the driving force for water uptake. AgNPs can induce the formation of nanopores in seeds, which helps absorb water, activates the antioxidant mechanism in seeds, generates hydroxyl free radicals to loosen cell walls, and induces seeds to enhance metabolic activity and starch hydrolysis—thereby increasing the water uptake rate of seeds [3,39], and promoting seed germination [40].

AgNPs can alter seed porosity, enhance the seed’s absorption of water and nutrients, and promote seed germination and seedling growth. For example, rice seeds treated with AgNPs were separated into seed coat, embryo, and endosperm; the results showed that Ag in the form of nanoparticles (rather than silver ions) was detected in all three parts. Therefore, when seeds absorb water through the small pores in the seed coat, AgNPs may pass through the seed coat and enter the interior along with the water flow [41]. In addition, after soaking rice seeds in AgNP suspensions at different concentrations (0.1, 1, 10, 100, 1000 mg/L) and particle sizes (20, 30–60, 70–120, 150 nm), post-germination analysis revealed that when treated with smaller particle sizes (20 nm) at concentrations of 100 and 1000 mg/L, the AgNP concentration in the rice root tissue was higher. This suggests that smaller particle sizes of AgNPs are more easily absorbed by the seeds and primarily accumulate in the roots rather than being transported to the leaves. Therefore, it can be concluded that when seeds are treated with smaller particle size AgNPs, the particles exhibit a stronger ability to penetrate into the plant roots, whereas larger particle sizes (70–120, 150 nm) can be detected in the aerial parts at a concentration of 10 mg/L [42]. This phenomenon may be related to seed porosity, which in turn affects the penetration and transportation of AgNPs. Data suggest that large-sized AgNPs can enter plant cells through endocytosis, or through non-endocytic pathways such as binding to carrier proteins, passing through aquaporins or ion channels, and inducing the formation of “new pores” on the cell membrane surface [42,43].

For instance, when sunflower seeds were treated with bimetallic ALAAgCuNPs, CuNPs with smaller particle sizes interacted with the seed coat to form small pores, and AgNPs further enlarged these pores, ultimately altering membrane fluidity [44].

The potential mechanism by which AgNPs affect seed germination is extremely complex; AgNPs can also change the water uptake kinetics of seeds, thereby influencing seed germination rate and survival rate. AgNPs release Ag^+^, which can bind to proteins or enzymes in the cell membrane. At high concentrations, Ag^+^ interferes with components such as aquaporins in the cell membrane. Impaired cell membrane function directly reduces the absorption and transportation of water in seeds and affects the osmotic state of seeds. Moreover, the activity of enzymes critical for seed germination (such as amylase and protease) inside the seeds is also inhibited by excessive Ag^+^, leading to metabolic disorders inside the seeds and exerting toxicity on the water uptake capacity of seeds [45].

## 4. Mechanisms of Action of AgNPs Affecting Seed Germination

### 4.1. Enhancing Seed Resistance to Pathogens

The long-term application of conventional antimicrobial agents in agricultural fields has led to the development and accumulation of drug resistance genes in various microorganisms, while the use of pesticide transformation products (PTPs) poses potential risks to non-target organisms. PTPs may exhibit greater persistence, mobility, and bioaccumulation potential compared to their parent pesticides, and excessive pesticide use threatens both ecosystems and human health. To mitigate these environmental hazards, nanotechnology has recently emerged as a promising alternative [46,47].

Plant diseases are common during plant growth; disease infection inhibits the normal growth and development of plants, leading to reduced crop yields and significant economic losses [48]. Specifically, diseases can trigger a series of adverse effects on the seed metabolic system, such as the downregulated expression of enzymes related to glycolysis and the tricarboxylic acid cycle (both involved in energy metabolism), the upregulation of heat shock proteins, antioxidant enzymes (AOEs), and defense-related enzymes (associated with stress response and defense), as well as changes in multiple processes including signal transduction, secondary metabolism, protein synthesis and storage, and cell structure and transport—all of which contribute to decreased crop yields [49]. Existing studies have shown that after soaking seeds in a certain concentration of AgNPs, the growth of plant pathogens *Alternaria solani*, *Alternaria alternata*, and *Colletotrichum gloeosporioides* was inhibited; compared with the antifungal drug amphotericin B (AmB), AgNPs exhibited better antifungal effects, and AgNPs at the minimum pathogen inhibitory concentration showed no toxic effects on seed germination [50]. Research indicates that AgNP pretreatment reduced the infection levels of two pathogenic fungi, *Didymella. pinodes* and *Fusarium*. avenaceum, on pea seedlings; after pea infection by fungi, the metabolite profiles of polar metabolites (e.g., phosphate, carbohydrates) in peas changed—especially the changes in carbohydrates and amino acids (AAS), which further altered secondary metabolites that play important roles in plant defense mechanisms, and among these changes, concentration variations in certain AAS (such as aspartic acid, homoserine, and proline) and carbohydrates (such as sucrose and glucose) may affect plant defense signaling pathways, ROS production, or the expression of defense-related proteins, thereby influencing the synthesis of secondary metabolites. However, after AgNP treatment, the metabolite content showed better stability, which enhanced the plant resistance and helped plants maintain normal metabolic functions after fungal infection [51].

**Figure 4:**
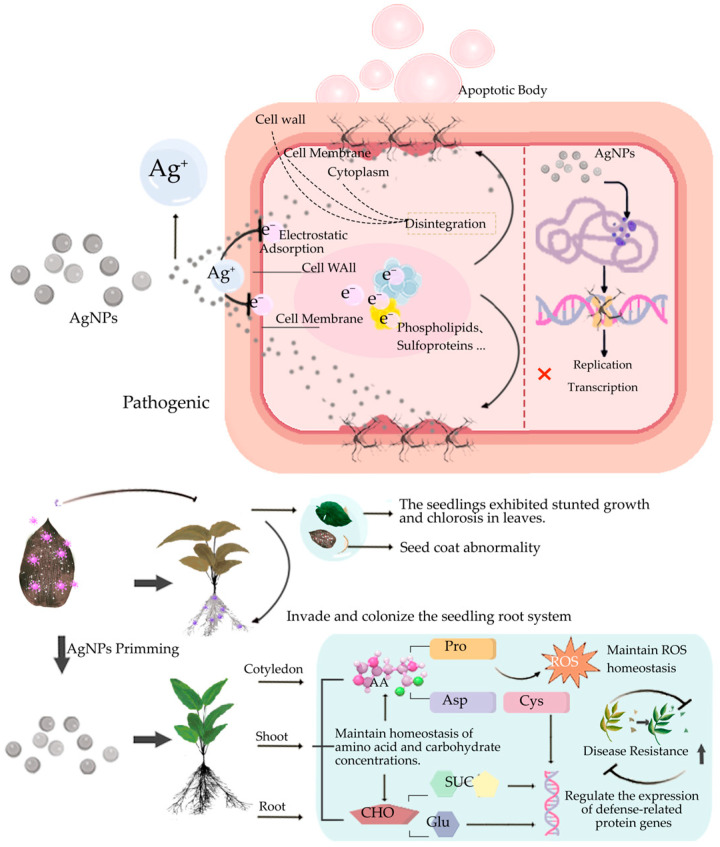
Schematic illustration of the mechanisms by which silver nanoparticle priming combats pathogens. AgNPs electrostatically adsorb to the pathogen’s cell membrane/wall, penetrate the cell, and bind DNA to block replication and transcription, suppressing pathogen proliferation. In seedlings, AgNP priming prevents seed-coat abnormalities and root invasion, alleviates stunting and leaf chlorosis, maintains amino-acid and carbohydrate homeostasis, scavenges excess ROS, and up-regulates defense-related genes, ultimately enhancing disease resistance. Amino Acid (AA); Proline (Pro); Aspartic Acid (Asp); Cysteine (Cys); Carbohydrate (CHO); Glutamic Acid (Glu); Sucrose (SUC). This figure was generated using Adobe Photoshop 2022.

Compared with seeds soaked in bovine submaxillary mucin (BSM) alone, BSM-treated pathogen cells showed typical rod shape with clear cell walls, while BSM-AgNP-treated cells exhibited disordered morphology, damage, surface vesicles, and intracellular AgNPs. Backscatter analysis revealed high atomic density materials on BSM-AgNP-treated cell surfaces, proving AgNPs binding to pathogens and consequent cell wall changes [52]. AgNPs significantly inhibit pathogen growth, biofilm formation, motility, and pathogenicity by severely damaging cell walls. Capping agent-stabilized AgNPs show enhanced antibacterial activity due to increased surface positive charges [25], enabling uniform dispersion [16]. Electrostatic interactions between bacterial cells and AgNPs facilitate AgNPs’ penetration into negatively charged pathogen cells [53], where released Ag^+^ disrupts membrane integrity, causing cytoplasmic and cell wall collapse [54], ultimately leading to pathogen apoptosis [55]. Although AgNPs enhance seed disease resistance, their interaction with beneficial microorganisms may reduce germination rates [5], warranting further research in this field (Figure 4). In summary, AgNPs significantly reduce pathogen-induced infection pressure on seeds through a three-step action chain of “physical damage–ion release–metabolic homeostasis,” thereby providing primary protection for subsequent germination.

### 4.2. Mechanisms of AgNP Effects on Seed Physiology and Biochemistry

**Figure 5:**
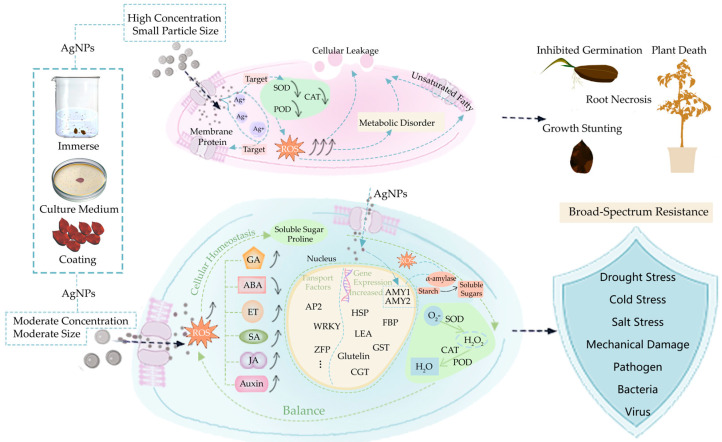
Schematic illustration of the mechanisms by which AgNP priming inhibits or promotes seed germination. After entering the seed, AgNPs modulate the levels of reactive oxygen species (ROS), which in turn trigger signaling pathways and regulate gene expression. AP2domain containing protein (AP2); Zinc finger proteins (ZFP); WRKY protein family (WRKY); Gibberellin (GA); Abscisic acid (ABA); Jasmonic acid (JA); Salicylic acid (SA); Ethylene (ET) AP2domain containing protein (AP2); Zinc finger proteins (ZFP); Late embryogenesis abundant protein (LEA); Glutathione S-transferase (GST); Heat shock protein (HSP); F-box protein (FBP); Cytokinin-O-glucosyltransferase (CGTs); Alpha-Amylase 1 (AMY1); Alpha-Amylase 2 (AMY2); Superoxide dismutase (SOD); Peroxidase (POD); Catalase (CAT). This figure was generated using Adobe Photoshop 2022.

AgNP priming of seeds can profoundly regulate seed germination, growth, and stress resistance by influencing ROS signaling, the antioxidant system, metabolic pathways, hormone balance, as well as gene and protein expression (Figure 5).

#### 4.2.1. Regulation of ROS Balance and Activation of the Antioxidant Defense System

ROS play a dual role in seed germination. As signaling molecules, at appropriate concentrations, ROS can initiate signaling transduction pathways such as GA and inhibit the ABA pathway, thereby promoting cell expansion and seed germination [56]. However, when seeds encounter drought, salinity, and low-temperature stresses, ROS levels rise sharply, subsequently inhibiting seed germination [57]. It is at this point that AgNPs become significant in enhancing antioxidant enzymes that scavenge ROS. Seeds of the winged bean were soaked in AgNPs at concentrations of 10, 50, 100, and 200 mg/L (with an average particle size of 15.82 nm). The activities of catalase, ascorbate peroxidase, and superoxide dismutase in the seeds were highest at an AgNP concentration of 50 mg/L, while a decreasing trend was observed at concentrations of 100 and 200 mg/L. The enhancement of these enzyme activities helps to scavenge excess ROS, avoid oxidative damage, and thereby promote germination and seedling growth [58]. When rice seeds were treated with AgNPs at concentrations of 10 mg/L and 20 mg/L (with an average particle size of 11.2 nm), the seeds treated with 20 mg/L exhibited higher catalase (CAT) activity and a higher germination rate compared to those treated with 10 mg/L [3].

In the *Solanum lycopersicum* L. seed germination experiment, AgNPs (with an average particle size of 20 nm) were applied at concentrations of 10, 20, 40, and 80 mg/L. At the 10 mg/L treatment, the activities of CAT, superoxide dismutase (SOD), ascorbate peroxidase (APX), and glutathione peroxidase (GPX) were significantly lower than the elevated levels observed at ≥40 mg/L. Meanwhile, the highest sugar and protein content was recorded, and germination was optimal. When the concentration was ≥40 mg/L, the activities of these four enzymes increased sharply, while nutrient content decreased drastically. Malondialdehyde (MDA) levels and membrane leakage rose significantly, leading to inhibited germination [59]. Therefore, the impact of AgNPs on seed germination is significantly concentration-dependent. Within the ‘signaling window’ of 10–50 mg/L, ROS induced by AgNPs primarily function as signaling molecules, activating the antioxidant enzyme system and scavenging excess ROS, thus promoting germination and seedling growth. Once the concentration exceeds this window, ROS quickly shift to become stress inducers, causing oxidative damage and inhibiting germination. Hence, the ‘signal-stress’ dual identity of AgNPs-ROS continuously transitions with dose, and precisely controlling the concentration is key to maintaining it within the beneficial signaling range.

Furthermore, the mixed ratios of AgNPs with other metal nanoparticles also have different effects on the antioxidant defense system. For example, at the same dosage, the metal ratio of AgCu and AgAu directly determines the strength of their stimulation on seed germination and antioxidant enzyme activity in *Eruca sativa* seeds. A higher Cu ratio (AgCu 1:3) results in the smallest particle size, the largest specific surface area, and the highest ion availability for interaction with plant cells, leading to the greatest toxicity [60]. In addition, the highest Cu^2+^ release occurs with the AgCu 1:3 ratio, leading to the strongest oxidative stress. This results in the highest activities of SOD and peroxidase (POD), but the germination rate drops to 80%, and seedling growth is inhibited. In contrast, higher Ag or Au ratios (AgCu 3:1, AuCu 1:3, AgAu 3:1) reduce toxicity, with germination rates reaching 100% and seedling vigor indices approaching those of the control, with AgCu 3:1 showing the best overall performance. Therefore, if the goal is high-yield cultivation, AgCu 3:1 should be selected. If the aim is to enhance medicinal compounds or induce stress tolerance, a short exposure to AgCu 1:3 may be used, but the dosage must be strictly controlled [61].

In conclusion, in addition to controlling the concentration of AgNPs, selecting the appropriate metal ratio based on the properties of the metals is crucial for optimizing seed germination and enhancing stress tolerance in crops.

#### 4.2.2. Activation of Key Metabolic Enzymes and Influence on Metabolic Pathways

AgNPs can affect various enzymes directly related to seed germination and energy supply. Studies have indicated that nanomaterials can increase the expression of the Alpha-Amylase 1 (AMY1) and Alpha-Amylase 2 (AMY2) genes, which control α-amylase activity [62,63]. For instance, the α-amylase activity in Ricinus communis L. seeds [64], Oryza sativa L. seeds [41], *Oryza sativa* L. [3], and *Triticum aestivum* L. [65] has been enhanced. Concurrently, the complex formed by AgNPs with α-amylase catalysis promotes starch hydrolysis to generate soluble sugars, mediates ROS production, thereby inducing cell wall loosening and facilitating seed germination [66,67]. Simultaneously, ROS synchronously activates the seed’s endogenous antioxidant enzyme defense system, manifesting as a significant upregulation in the activities of key enzymes such as SOD, CAT, and POD, which finely regulate and maintain ROS level balance, thereby collaboratively completing the process of cell wall remodeling and germination initiation [41,64].

However, AgNPs may also inhibit certain key metabolic enzymes. For example, AgNPs can bind to thiol groups in proteins, leading to decreased activity of enzymes such as glyceraldehyde-3-phosphate dehydrogenase (GAPDH) and malate dehydrogenase (MDH), both of which are crucial for seed metabolism and functional regulation [68,69,70]. Proteomic analysis of wheat indicated that AgNP priming can induce an increase in proteins related to protein synthesis while decreasing proteins associated with glycolysis, signaling, and the cell wall. Proteins related to redox processes and the mitochondrial electron transport chain were also reduced. Specifically regarding glycolysis-related proteins, the expression of glyceraldehyde-3-phosphate dehydrogenase showed both increases and decreases, whereas phosphoenolpyruvate carboxylase was reduced. This suggests that AgNPs may regulate energy metabolism by inhibiting glycolysis, thereby diverting more resources to other growth processes such as protein synthesis, ultimately promoting germination and development [65].

AgNPs support energy supply and cell wall remodeling by activating key enzymes, such as α-amylase and antioxidant enzymes, while simultaneously inhibiting other enzymes involved in glycolysis. This resource redistribution facilitates seed germination and development. These effects highlight the dual role of AgNPs in seed germination, exerting both promotive and inhibitory influences.

#### 4.2.3. Induction of Metabolite Accumulation and Reprogramming

Metabolites are fundamental to plant life activities, and their changes profoundly influence growth, development, and stress resistance. Research has shown that AgNPs can significantly alter the seed metabolome, with induced metabolite accumulation highly correlated with the morphological characteristics of AgNPs (such as particle size and surface area). Studies have indicated that the surface area and particle size of nanoparticles influence the content of total flavonoids and total phenols. Smaller nanoparticles (15–20 nm) may induce more biological stress, thereby promoting the production of secondary metabolites in seedlings [61]. Moreover, the impact of AgNPs on seed metabolism is concentration-dependent. Seven concentrations of AgNP solutions (5, 10, 15, 20, 25, 50 mg/L) were tested, and it was found that a 10 mg/L concentration of AgNPs significantly increased the levels of chlorophyll, carotenoids, alkaloids, flavonoids, and soluble sugars in tomato plants. This enhanced photosynthetic efficiency, energy supply, osmotic regulation, defense mechanisms, and antioxidant capacity [71].

Another study on the effect of AgNPs on pea seed germination found that the metabolic reprogramming induced by AgNPs is also concentration-dependent. The experiment set up seven concentrations (25, 50, 75, 100, 150, 200 mg/L). At a concentration of 50 mg/L, AgNPs enhanced respiratory metabolism (such as the accumulation of malate and phosphates) while reducing sucrose content in the roots and increasing sucrose in the hypocotyl, directing carbon sources to the growth zones. Proline accumulated significantly, enhancing osmotic and antioxidant protection, indicating positive metabolic adaptation. However, at a concentration of 200 mg/L, AgNPs exhibited inhibitory metabolic characteristics: intermediates in the tricarboxylic acid cycle decreased, suggesting restricted respiration, while monosaccharides accumulated in the hypocotyl but with reduced utilization efficiency. Nitrogen transport was hindered, proline’s protective ability weakened, and γ-aminobutyric acid accumulated. The metabolic response showed signs of disruption, indicating that the metabolic effects of AgNPs are concentration-dependent. Notably, at concentrations of 50 mg/L and 200 mg/L, AgNPs did not significantly inhibit pea seed growth. In contrast, the same concentrations of AgNPs significantly inhibited the growth of *Triticum aestivum* L. and *Lepidium sativum*, while in radishes, the effect was characterized by low-concentration (75 mg/L) promotion and high-concentration (150–200 mg/L) inhibition. This suggests that the effects of AgNPs are species-dependent [72].

At the same time, other studies have shown that the impact of AgNPs on seed metabolism is genotype-dependent. For example, when aging-sensitive inbred line I178 and aging-tolerant inbred line X178 maize seeds were soaked in a 1.08 mg/L AgNP solution, the transcriptome of seedlings from the aging-sensitive inbred line I178 showed that AgNPs significantly upregulated genes related to antioxidant and redox metabolism, lipid metabolism, and membrane integrity. It also induced pathways related to carbohydrate metabolism, fatty acid β-oxidation, phenylpropanoid biosynthesis, and cell wall synthesis, thereby repairing membrane damage, scavenging reactive oxygen species, and reallocating energy. However, under the same treatment, these pathways were not significantly induced in the aging-tolerant inbred line X178, indicating that this metabolic reprogramming effect is genotype-dependent [73].

#### 4.2.4. Regulation of Plant Hormone Balance

Hormones play a central regulatory role in seed germination and stress responses. AgNP priming can promote germination and enhance environmental adaptability by modulating hormone levels. For example, AgNP treatment can increase GA content and decrease ABA content. The balance between GA and ABA precisely regulates the breaking of seed dormancy [74], thereby promoting seedling growth and increasing plant height. Specifically, the content of gibberellin G6 increases significantly while cytokinin content decreases. This shift in ratio facilitates the transition from vegetative to reproductive growth in crops, promoting earlier flowering and increasing yield [75]. Simultaneously, AgNP treatment can also elevate the levels of jasmonic acid (JA) and salicylic acid (SA), and the increase in these two hormones can enhance plant immunity against stress [75,76]. AgNP pretreatment can significantly increase ROS levels in the embryo tissue of seeds, participating in the regulation of complex hormone signaling pathways [77].

In earlier research, it was suggested that NPs might mimic Ca^2+^, “deceiving” calcium-binding proteins and subsequently activating them [78]. However, a more recent study on rice seeds refutes this claim. It indicates that after AgNPs penetrate the seed, they mimic peroxidases, catalyzing ROS production. Acting as an upstream “signal messenger,” ROS activates the calcium signaling pathway (CDPKs) and multiple plant hormone signaling networks. This activation strengthens the signaling of germination-related hormones such as auxin, GA, and ethylene, as well as stress-responsive hormones like SA, ABA, and JA [41].

Overall, AgNPs enhance ROS levels within the seed embryo, triggering a series of hormone signaling pathways related to germination, thereby promoting the breaking of seed dormancy and the initiation of germination. Simultaneously, AgNPs increase the levels of jasmonic acid and salicylic acid, boosting the plant’s immune response, and regulate the plant’s stress response and rapid seedling emergence by downregulating ABA content. These changes form a coordinated signaling network that promotes rapid seed germination and environmental adaptability.

### 4.3. Molecular Network Impact Mechanisms of AgNPs on Seed Gene Expression and Proteome

AgNP priming can induce widespread changes in gene expression at the transcriptional level. For example, after tobacco seeds were treated with AgNPs, (1.00 μg/mL, 10–20 nm, nearly spherical), genes related to glutathione-sulfur metabolism, alanine-aspartate-glutamate metabolism, and cell wall loosening were significantly upregulated in the root tissue. In the leaf tissue, genes associated with carotenoid biosynthesis were upregulated, while specific ABA-related genes showed differential expression. The activity of certain peroxidases in the roots decreased. These coordinated changes enhanced the overall antioxidant capacity, photosynthetic efficiency, and root elongation of the plants, manifesting as faster germination, uniform seedling emergence, increased root length, higher chlorophyll and carotenoid content, and an increase in the maximum quantum efficiency of photosystem II (Fv/Fm). Meanwhile, the damage caused by environmental stress to tobacco was alleviated, leading to a significant increase in both fresh and dry weights of the plants [79].

Simultaneously, the molecular effects of AgNP priming are also reflected in proteomic changes, as revealed by proteomic analyses. During the water imbibition stage before seed germination, AgNP priming can promote greater water uptake by seeds, thereby up-regulating the expression of aquaporin genes and improving seed germination rate and quality [3,80]. A study on wheat seeds found that AgNP priming altered the protein expression profile: it induced an increase in proteins related to protein synthesis, reduced proteins associated with glycolysis, signaling, and the cell wall, and also decreased proteins related to redox processes and the mitochondrial electron transport chain [65]. Soaking tobacco seeds with AgNP treatment during the imbibition stage led to changes in 58 protein groups. Among these, proteins related to photosynthesis, antioxidant defense, protein synthesis and folding, and amino acid metabolism were up-regulated, while proteins involved in RNA processing were also altered. These changes collectively enhance antioxidant defense capacity and adaptability to silver-induced stress [81,82]. In other words, AgNPs enhance defense responses, accelerate water uptake, improve stress tolerance, and promote germination and development by regulating the gene expression and proteome of seeds and seedlings.

### 4.4. Regulation of the Seed Germination Microenvironment

Soil pH is a critical factor influencing seed germination, as it significantly affects soil fertility and various biological, chemical, and physical properties and processes related to plant growth and biomass yield [83]. The soil pH value not only alters nutrient availability by affecting soil chemical reactions but also regulates plant root nutrient absorption rates, consequently impacting the uptake of metal ions such as zinc [84].

Experiments demonstrated that adding 100 mg/kg AgNPs to both planted and unplanted cucumber soils significantly increased soil pH, likely due to AgNP-induced alterations in soil metabolite composition [85]. Furthermore, AgNP inhibition of soil urease, sucrase, and respiration showed positive correlation with soil pH. It is important to note that silver ions released from AgNPs tend to form Ag_2_CO_3_ under high pH conditions, while potentially forming AgCl in low pH environments. These pH-dependent chemical speciation changes affect the solubility, bioavailability, and subsequent toxicity of Ag^+^ [86]. Additional experiments revealed concentration-dependent inhibition of soil enzyme activity by AgNPs, with higher concentrations causing greater enzymatic reduction [87]. As soil enzymes play crucial roles in organic matter decomposition, their suppressed activity impedes this process [88], which normally generates organic acids that influence soil pH [89].

The toxicity of AgNPs to soil microorganisms is closely related to soil physicochemical properties, including pH, organic matter content, and clay distribution, which collectively influence the aggregation state and stability of AgNPs in soil. Studies show that AgNP-induced inhibition of soil respiration, urease, and sucrase activities positively correlates with soil pH, with greater biomass impacts observed in high-pH soils. Acidic soils exhibit lower surface electronegativity and reduced electrostatic repulsion against AgNPs, facilitating their binding to soil particles and consequently decreasing AgNPs mobility. As a result, AgNPs demonstrate stronger toxicity in acidic soils compared to alkaline conditions, exerting more pronounced effects on seed germination and development [90].

Soil microbial status serves as a crucial indicator for assessing soil quality, with the interactions between AgNPs and soil microorganisms/enzymes significantly influencing plant growth and quality [91,92]. Due to their unique physicochemical properties, AgNPs readily interact with microbial surfaces, and given the marked differences in microbial sensitivity to AgNPs, they can alter the structure of various soil microbial communities, including the relative abundance of Acidobacteria, Verrucomicrobia and Proteobacteria [93], Actinobacteria, Cyanobacteria, Nitrospirae, and Planctomycetes [94]. A study simulating the release of Ag^+^ from AgNPs in soil using AgNO_3_ revealed differences in soil sensitivity. The half-maximal effective concentration (EC_50_) values for soil microbial respiration (SIR) across nine Australian soils ranged from 3.3 mg/kg to 434.7 mg/kg, demonstrating significant soil-dependent variability. The positive correlation between SIR and bacterial 16S rRNA gene copy numbers suggests that microbial respiration activity relates to bacterial abundance changes. Ag^+^ released from AgNPs strongly inhibits bacterial community abundance but shows lesser effects on fungal communities, likely due to fungal cell walls providing better protection against Ag^+^ toxicity [86]. Furthermore, microbial abundance changes correlate with their inherent AgNP tolerance—for instance, AgNPs increase the relative abundance of Glomeraceae while decreasing Acaulosporaceae in AM fungal communities. This shift may occur because Glomeraceae fungi more efficiently utilize recently fixed plant carbon, gaining competitive advantages and greater AgNP tolerance [95]. Additionally, AgNPs can modify microbial community structure and function to influence soil ecology, selectively enriching specific nitrogen-fixing bacteria and optimizing microbial composition to create favorable conditions for soil nitrogen fixation. However, AgNPs exert multifaceted effects on soil nitrogen transformation: they reduce soil microbial biomass and nitrogen-fixing bacteria numbers, potentially limiting nitrogen availability for plants and soil fertility maintenance, while also decreasing leucine aminopeptidase activity and impairing soil biochemical functions [96].

Studies have revealed that AgNPs can influence various soil enzyme activities, including leucine aminopeptidase (LAP), soil dehydrogenase (SDHA), sulfatase, β-glucosidase, and urease, with their dose, coating, particle size, and exposure time being critical determining factors [97,98]. The released Ag^+^ from AgNPs may bind to enzyme active sites, adsorb onto enzyme surfaces, or interact with other soil components, thereby disrupting normal enzymatic functions [96]. The interactions between soil enzymes and AgNPs partly depend on their surface charges: uncoated smaller AgNPs release more Ag^+^ and have greater contact area with enzymes, while coatings modify surface charges—citrate-AgNPs carry stronger negative charges than PVP-AgNPs, reducing direct enzyme contact. Moreover, PVP coating enhances AgNPs dispersibility and reactive surface components, whereas citrate coating may stimulate certain enzymes, making citrate-AgNPs generally less toxic than uncoated or PVP-AgNPs. Notably, soil enzymes exhibit stronger inhibition even under low PVP-AgNP exposure [98]. Different coatings show distinct inhibitory patterns: PEI-coated AgNPs most strongly suppress urease activity, while citrate-coated ones predominantly inhibit dehydrogenase activity. Such enzyme inhibition intensifies with increasing AgNP concentrations but weakens over time, suggesting possible microbial adaptation to nanoparticle toxicity [99].

### 4.5. The Effects of Ag^+^ Ions and AgNPs on Germination

The release of Ag^+^ from AgNPs is one of the key pathways through which AgNPs affect seed germination, but the absorption pathways and modes of action for the two differ fundamentally. When wheat seeds were placed on filter paper containing 20 mg/L citrate-coated AgNPs (10–100 nm) for 3 days, TEM-EDS (transmission electron microscopy–energy dispersive spectroscopy) observations revealed that only smaller particles could enter through the root epidermal cell wall pores, while particles ≥ 60 nm were mostly blocked [3]. In contrast, Ag^+^ at the same concentration (20 mg/L) instantly penetrated the cell wall in its ionic form, without size limitations [100]. This indicates that the Casparian strip acts as a barrier to particulate Ag, whereas ionic Ag can freely cross and accumulate rapidly in the shoot. Additionally, AgNPs induce localized ROS bursts and spotty cell death in the root tip meristematic and elongation zones, with sucrose levels increasing, monosaccharides decreasing, and the total amino acids and organic acids remaining relatively unchanged. Ag^+^, however, causes uniform oxidative stress throughout the root and broadly inhibits energy and nitrogen metabolism [37,101,102].

When tomato seeds were soaked in soluble AgNO_3_ and green-synthesized AgNPs (average particle size 20 nm, spherical), AgNPs at 10 mg/L slightly promoted tomato germination and increased sugar and protein content, while Ag^+^ at the same concentration significantly inhibited germination. At 80 mg/L, AgNPs caused significantly less membrane leakage, lipid peroxidation, and decreases in protein/sugar content compared to Ag^+^, with a moderate increase in antioxidant enzymes. The slow release of Ag^+^ by AgNPs raises its toxicity threshold, expanding the safe growth window approximately 4 times compared to soluble silver sources, which also demonstrates the advantage of AgNPs over Ag^+^ in terms of low-dose efficiency and high-dose low toxicity [59].

In parallel comparison experiments with AgNO_3_ solution as a silver ion control, wheat seeds soaked in green AgNPs showed that at the same soaking concentrations (10–80 mg/L), AgNO_3_ also slightly increased plant height, yield, and grain NPK, but the increases were significantly lower than those of AgNPs. When the concentration was raised to 80 mg/L, both treatments showed a decline, but the decrease was more gradual with AgNO_3_. The authors noted that Ag^+^ is easily fixed in the soil, causing its effective concentration to decrease rapidly and its action to be brief, while AgNPs can slowly release Ag^+^ and be directly absorbed by seeds/roots, dual-regulating physiological processes, thus resulting in better growth-promoting effects compared to pure silver ion treatments [103].

## 5. Existing Problems and Controversies

### 5.1. Limitations in Mechanistic Studies

Although current research on the mechanisms through which AgNPs affect seed germination has made some progress, several critical limitations remain to be addressed. In terms of exposure systems, most existing studies rely on hydroponic setups. While such systems allow for precise control of AgNP concentration, pH, dissolved oxygen, and other parameters—thereby simplifying variable interference and enabling clear observation of the direct interaction of AgNPs with seed germination—they significantly differ from real soil environments. In natural soil, AgNPs undergo adsorption processes with clay minerals, humic acids, and other components. Their aggregation, dissolution, and transformation are regulated by factors such as soil microbial communities and redox potential, which alter the bioavailability and toxicity of AgNPs [104,105]. While soil-based and artificial substrate experiments more closely mimic natural conditions, they often face challenges in accurately quantifying AgNP exposure doses. Moreover, significant differences in physicochemical properties across soil types—such as acidic red soil versus alkaline brown soil—further reduce the comparability of experimental results.

In terms of research subjects and generalizability, studies have predominantly focused on model plants like rice or common crop species. Research on economic crops, medicinal plants, ornamental species, and endangered plants remains limited [106]. Different plant species exhibit significant variations in sensitivity and response mechanisms to AgNPs [107], making it difficult to extrapolate existing findings to a broader range of plant groups. This limitation hinders the application of AgNPs in diverse agricultural contexts.

### 5.2. Interactions Between Hormones, Nutrient Elements, and Environmental Factors

In the investigation of how AgNPs affect seed germination, the interactions among environmental factors are also particularly important. However, the mechanisms by which AgNPs synergistically promote seed germination in combination with other substances remain unclear. Most existing studies have focused on the effects of individual substances, while the potential synergistic effects between AgNPs and plant hormones, biostimulants, or nutrient elements such as nitrogen, phosphorus, and potassium have not received adequate attention [108]. These combinations of substances may collectively promote seed germination by regulating seed respiratory metabolism, hormone balance, and the antioxidant system. Nevertheless, systematic studies are currently lacking to elucidate the molecular mechanisms underlying their combined effects and to determine the optimal ratios for such interactions [109].

Furthermore, research on the effects of AgNPs under multiple external environmental stress conditions remains notably insufficient. In agricultural practice, plants are often exposed to diverse and complex stresses, which may alter the influence of AgNPs on seed germination through synergistic, antagonistic, or additive interactions. However, most current studies focus solely on the impact of individual stresses and lack analysis of the interactions under combined stress scenarios. This limitation hinders the development of reliable risk assessment models and consequently constrains the safe application and scientific management of AgNPs in agricultural ecosystems.

### 5.3. Insufficient Ecological Risk Assessment

Regarding long-term cumulative effects, current studies predominantly focus on the short-term impacts of AgNPs on seed germination, typically through acute exposure experiments lasting 1 to 7 days, while research on their long-term cumulative effects remains significantly limited [110]. For instance, field studies have shown that AgNPs can reduce rice diseases and increase yields by approximately 30%, directly contributing to food security. However, the same study also detected silver residue in the topsoil, with a dose of 0.01 mg/kg significantly inhibiting nitrogen-fixing bacterial activity and microbial diversity after one year. This suggests that long-term accumulation could damage soil ecosystems and transfer through the food chain. Therefore, while pursuing yield improvements, it is essential to implement degradable formulations and strict environmental threshold management [111]. After entering the soil, AgNPs may gradually accumulate through processes such as adsorption and complexation. It remains unclear whether continuous exposure of seeds to AgNPs over multiple generations could result in progressive accumulation of silver in seed tissues such as the seed coat and endosperm, thereby affecting the viability of progeny seeds—including germination rate, germination vigor, and seedling growth rate. Furthermore, systematic multi-generational experimental data are currently lacking to determine whether long-term exposure may induce genotoxicity (e.g., gene mutations, chromosomal aberrations) in plants, or whether such alterations could be transmitted to offspring through seeds [112]. However, the few existing field trials have only focused on the current season’s yield and physiological indicators, without tracking soil residue, subsequent season accumulation, or transgenerational effects. There is still a significant gap in risk assessments at the level of non-target organisms and ecosystems [113].

There remains a substantial gap in risk assessment concerning non-target organisms and ecosystem-level impacts. The antimicrobial properties of AgNPs may adversely affect soil microbial communities (e.g., nitrogen-fixing bacteria, mycorrhizal fungi), soil fauna, and aquatic organisms, thereby disrupting material cycles and energy flows within ecosystems [114]. However, related research remains extremely limited. For instance, the long-term effects of AgNPs on the structure and functional genes of soil microbial communities, such as those involved in nitrogen and carbon metabolism, have not yet been fully elucidated. Moreover, the potential risks of AgNP transmission through the food chain to higher trophic-level organisms remain inadequately evaluated.

## 6. Future Research Directions

In future studies on the mechanisms through which AgNPs influence seed germination, it is necessary to move beyond the limitations of traditional population-level cellular analysis and advance toward single-cell resolution and dynamic tracking. Utilizing technologies such as spatial transcriptomics and single-cell metabolomics will enable the analysis of spatiotemporal changes in gene expression and metabolites within seed cells at the single-cell level following AgNP treatment. This will facilitate the creation of cellular response profiles under AgNP-induced stress. Notably, deep learning algorithms can be employed to analyze high-throughput gene expression data. By constructing gene regulatory network models, these approaches can predict potential AgNP-responsive genes and their upstream–downstream regulatory relationships, providing new theoretical insights for mechanistic research.

Meanwhile, future research needs to integrate materials science, environmental science, and bioinformatics to establish a multidimensional database containing the physicochemical properties of AgNPs (e.g., particle size, surface charge, surface modification, crystal structure), environmental parameters (e.g., pH, temperature, ionic strength, organic matter content), and plant physiological parameters (e.g., antioxidant enzyme activity, hormone levels, gene expression). By leveraging machine learning algorithms and artificial intelligence technologies, complex interrelationships among the data can be mined, enabling the construction of high-precision predictive models. This will ultimately allow for accurate predictions of the toxic effects of different types of AgNPs.

## Data Availability

No new data were created or analyzed in this study. Data sharing is not applicable to this article.
